# Eligibility for PCSK9 treatment in 734 Hypercholesterolemic patients referred to a regional cholesterol treatment center with LDL cholesterol ≥70 mg/dl despite maximal tolerated cholesterol lowering therapy

**DOI:** 10.1186/s12944-016-0227-2

**Published:** 2016-03-12

**Authors:** Charles J. Glueck, Parth Shah, Naila Goldenberg, Marloe Prince, Kevin Lee, Vybhav Jetty, Ashwin Kumar, Michael Goldenberg, Ping Wang

**Affiliations:** From the Cholesterol, Metabolism, and Thrombosis Center, Jewish Hospital of Cincinnati, Cincinnati, USA

**Keywords:** PCSK9 inhibitors, LDL cholesterol (LDLC), Heterozygous familial hypercholesterolemia (HeFH), Atherosclerotic cardiovascular disease (CVD)

## Abstract

**Background:**

LDL cholesterol (LDLC) lowering has been revolutionized by PCSK9 inhibitors, Alirocumab (Praluent) and Evolocumab (Repatha), approved as adjuncts to maximally tolerated cholesterol lowering therapy in heterozygous (HeFH) or homozygous (HoFH) familial hypercholesterolemia, and/or clinical atherosclerotic cardiovascular disease (CVD) where LDLC lowering is insufficient.

**Methods:**

We applied FDA and insurance eligibility criteria for PCSK9 inhibitor use in 734 hypercholesterolemic patients serially referred over 3 years who then received ≥ 2 months maximally tolerated LDLC lowering therapy with follow up LDLC ≥ 70 mg/dl, and in 50 patients approved by insurance for PCSK9 inhibitors. We documented the percentage of patients with HeFH and/or CVD who met FDA and insurance criteria for PCSK9 inhibitor therapy using LDLC goal-based guidelines.

**Results:**

Of 734 patients with LDLC ≥ 70 mg/dl after ≥ 2 months maximally tolerated LDLC lowering therapy, 220 (30 %) had HeFH and/or CVD with LDLC > 100 mg/dl, meeting FDA-insurance criteria for PCSK9 inhibitor therapy. Another 66 (9 %) patients were statin intolerant, without HeFH or CVD. Of the 50 patients whose PCSK9 inhibitor therapy was approved for insurance coverage, 45 (90 %) had LDLC > 100 mg/dl after ≥ 2 months on maximally tolerated LDLC lowering therapy. Seventeen of these 50 patients (34 %) had HeFH without CVD (LDLC on treatment 180 ± 50 mg/dl), 15 (30 %) had CVD without HeFH (LDLC on treatment 124 ± 26 mg/dl), 14 (28 %) had both HeFH and CVD (LDLC on treatment 190 ± 53 mg/dl), and 4 (8 %) had neither HeFH nor CVD (LCLC 142 ± 11 mg/dl).

**Conclusion:**

Of 734 patients referred for LDLC reduction, with LDLC ≥ 70 mg/dl after ≥ 2 months on maximally tolerated therapy, 220 (30 %) had HeFH and/or CVD with LDLC > 100 mg/dl, meeting FDA-insurance criteria for PCSK9 inhibitor therapy as an adjunct to diet-maximally tolerated cholesterol lowering therapy in HeFH or CVD. If 30 % of patients with high LDLC and HeFH-CVD are eligible for PCSK9 inhibitors, then specialty pharmaceutical pricing models (~$14,300/year) will collide with tens of millions of HeFH-CVD patients. We speculate that if there was a 50 % reduction in CVD, then there would be savings of $245 billion, in the middle of the range of estimated PCSK9 inhibitor costs of $185-342 billion. Whether the health care savings arising from the anticipated reduction of CVD events by PCSK9 inhibitors justify their extraordinary costs in broad population use remains to be determined.

## Background

Lowering of LDL cholesterol (LDLC) has been revolutionized by the recent release of the PCSK9 inhibitors, Alirocumab (Praluent) and Evolocumab (Repatha). PCSK9 inhibitors are approved for patients with heterozygous familial hypercholesterolemia (HeFH), homozygous familial hypercholesterolemia (HoFH), and for patients with atherosclerotic cardiovascular disease (CVD) unable to achieve LDLC goals despite maximal tolerated cholesterol-lowering therapy, including zero dose tolerance (statin intolerance) [1–3]. Preliminary results of safety-efficacy controlled clinical trials, although not powered or designed to definitively assess CVD events, showed about a 50 % risk reduction in CVD events [1, 2].

If the annual cost of the PCSK9 inhibitors were to remain at $14,000–14,600, then specialty pharmaceutical pricing models previously reserved for drugs which benefitted limited patient populations will collide with prospective treatment cohorts in the tens of millions of patients at high risk for CVD, when using PCSK9 inhibitors as an adjunct to diet-maximally tolerated cholesterol lowering therapy in heterozygous (HeFH) or homozygous (HoFH) familial hypercholesterolemia, or clinical atherosclerotic cardiovascular disease (CVD). We have applied FDA approved and commercial insurance eligibility criteria for PCSK9 inhibitor use in 734 patients serially referred to our Cholesterol Diagnosis and Treatment center and receiving ≥ 2 months maximally tolerated LDLC lowering diet-drug therapy with follow up LDLC ≥ 70 mg/dl, to obtain estimates of the percentage of patients with HeFH and CVD who meet FDA and commercial insurance eligibility for PCSK9 inhibitor treatment using LDLC goal-based guidelines [4, 5].

## Methods

The study followed a protocol approved by the Jewish Hospital Institutional Review Board (JH #12–03).

We assessed 734 hypercholesterolemic patients consecutively referred to our Cholesterol Treatment Center from May 2012 to September 2015, who subsequently received ≥ 2 months of maximally tolerated diet-drug LDLC lowering therapy, with last follow up LDLC ≥ 70 mg/dl. All patients were instructed to consume a cholesterol lowering diet by a registered dietitian, and received maximally tolerated LDLC lowering therapy, predominantly with statins and a small percentage were also taking ezetimibe and/or colesevelam.

We assessed 50 patients who were approved for PCSK9 inhibitor therapy with Evolocumab or Alirocumab coverage by their medical insurance programs by applying the Simon Broome criteria for HeFH [6] and/or CVD with LDLC above target (> 100 mg/dl [4]) despite maximally tolerated LDLC lowering therapy.

After an overnight fast, lipids and lipoprotein cholesterols were serially measured by LabCorp with direct measurement of LDLC if triglycerides were > 400 mg/dl. Heterozygous familial hypercholesterolemia (HeFH) was defined by LDLC ≥ 190 mg/dl and the presence of tendon xanthomas and/or by hypercholesterolemic first degree relatives (Simon Broome criteria [6]). Atherosclerotic cardiovascular disease (CVD) included medical record-physician referral documented coronary artery, carotid, aortic, or peripheral vascular atherosclerosis, as well as transient ischemic attack and ischemic stroke. Patients intolerant to ≥ 3 statin medications were identified as “statin intolerant.”

Separately, we assessed fasting lipid profiles in 8053 patients at their first visit, consecutively referred to our center over the last 30 years for diagnosis and treatment of hypercholesterolemia. The 734 patients in the current report studied after ≥ 2 months on maximal lipid lowering therapy were a subset of these 8053 patients.

## Results

**Fig. 1:**
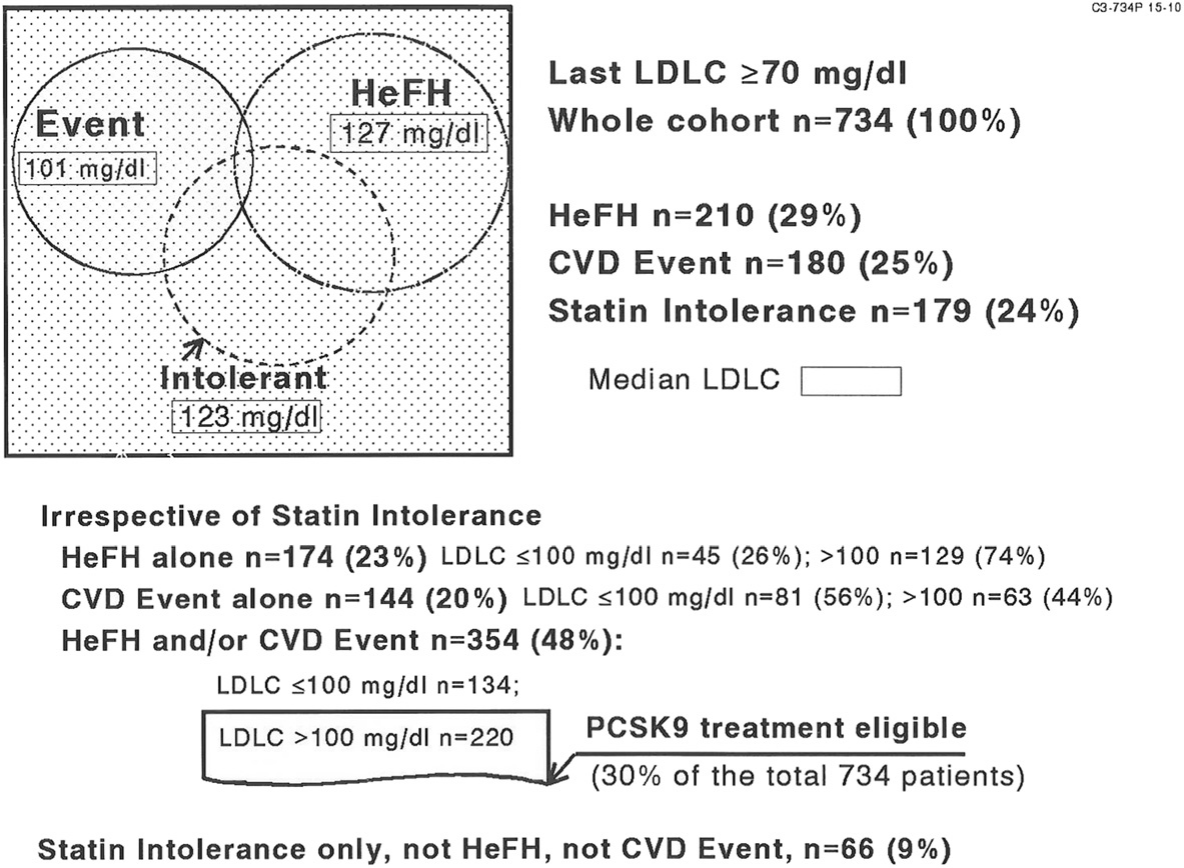
Heterozygous familial hypercholesterolemia (HeFH) and atherosclerotic cardiovascular events (CVD) in 734 patients with LDLC ≥70 mg/dl after ≥ 2 months maximally tolerated cholesterol-lowering therapy. Two hundred and twenty patients with HeFH and/or CVD (30 % of the referred cohort) eligible for PCSK9 inhibitor therapy by FDA and commercial insurance guidelines by virtue of LDLC on maximal therapy >100

Of the 734 patients, 210 (29 %) had HeFH, 180 (25 %) CVD, and 179 were statin intolerant (24 %), Fig. 1. In the 180 patients who had CVD, the first CVD event was at age 55 ± 13, median 55 years. Of the 734 patients, 66 (9 %) were statin intolerant, without HeFH or CVD, Fig. 1. After ≥ 2 months on maximum tolerated LDLC lowering therapy, median LDLC in patients with HeFH was 127 mg/dl, in those with CVD events, 101 mg/dl, and in statin intolerant patients, 123 mg/dl, Fig. 1.

Two hundred and twenty of the 734 patients (30 %) were eligible by both FDA and commercial insurance criteria for PCSK9 inhibitor treatment, by virtue of having HeFH and/or CVD, and also having LDLC > 100 mg/dl on maximally tolerated lipid lowering therapy, Fig. 1. Of these 220 patients, 161 had taken statins, 6 statins plus colesevelam, 3 colesevelam only, 11 statins plus ezetimibe, and 4 statins plus colesevelam and ezetimibe. Of these 220 patients, 56 (25 %) were unable to take any statins, being intolerant to ≥ 3 different statins.

Of the 220 patients having HeFH and/or a CVD event with LDLC > 100 mg/dl on maximal tolerated cholesterol lowering treatment (Fig. 1), 129 had HeFH alone, with LDLC on therapy 160 ± 51, median 147 mg/dl, 63 had CVD events alone, with LDLC on therapy 134 ± 29, median 121 mg/dl, and 28 had both HeFH and CVD, with LDLC on therapy 178 ± 46, median 174 mg/dl. In those 91 patients with CVD, the first CVD event occurred at age 54 ± 13, median 56 years.

Fifty patients were approved for PCSK9 inhibitor therapy (Evolocumab or Alirocumab) coverage by their medical insurance programs. Forty-five of these 50 patients (90 %) had LDLC > 100 mg/dl after ≥ 2 months on maximally tolerated LDLC lowering therapy, while in the other 5 patients, LDLC was 80–100 mg/dl. Seventeen of the 50 patients (34 %) had only HeFH but no CVD, with LDLC on treatment 180 ± 50 mg/dl, median 182 mg/dl. Fifteen of the 50 patients (30 %) had only CVD but no HeFH, with LDLC on treatment 124 ± 26 mg/dl, median 131 mg/dl, 14 of the 50 (28 %) had both HeFH and CVD, with on-treatment LDLC 190 ± 53 mg/dl, median 191 mg/dl, and the other 4 (8 %) patients had no HeFH nor CVD, with LDLC 129, 141, 141, 156 mg/dl. The patients with CVD had their first CVD event at age 56 ± 11, median 58 years.

Upon evaluation of initial visit LDLC levels in 8053 patients referred to our center over the last 30 years, 9 % had LDLC ≥190 mg/dl, 12 % fell between 160 and 190 mg/dl, 20 % between 130 and 160 mg/dl, 28 % between 100 and 130 mg/dl, and 31 % had LDLC < 100 mg/dl, Tables 1 and 2. Overall, 41 % of our 8053 referred patients had LDLC ≥ 130 mg/dl at their first referral visit, Tables 1 and 2. If LDLC ≥ 160 mg/dl is used as a cutoff for identification of hypercholesterolemic patients deserving treatment, 21 % of our 8053 patients had LDLC ≥ 160, and if LDLC of 190 is used as a treatment cutoff, 9.1 % were above this level, Tables 1 and 2.

**Table 1:** The distribution of low density lipoprotein cholesterol (LDLC) in 8053 patients serially referred to the Cincinnati Cholesterol Center for diagnosis and therapy of hypercholesterolemia

Categorized by triglyceride levels	LDLC<100 mg/dl	100≤LDLC<130	130≤LDLC<160	160≤LDLC<190	LDLC≥190 mg/dl	Whole row
						Percent of row
TG<200 mg/dl	1719 (68 %)	1567 (71 %)	1108 (68 %)	617 (64 %)	446 (61 %)	5457 (68 %)
200≤TG<250	198 ( 8 %)	230 (10 %)	190 (12 %)	126 (13 %)	111 (15 %)	855 (11 %)
250≤TG<300	150 ( 6 %)	132 ( 6 %)	115 ( 7 %)	85 (9 %)	83 (11 %)	565 (7 %)
TG≥300 mg/dl	445 (18 %)	292 (13 %)	215 (13 %)	134 (14 %)	90 (12 %)	1176 (15 %)
Whole Column	2512	2221	1628	962	730	8053 (100 %)
Percent of column	(31 %)	(28 %)	(20 %)	(12 %)	(9 %)	(100 %)

**Table 2:** LDLC percentiles in the 8053 patients

LDLC percentiles	Mean ± SD	2.5^th^ percentile	5^th^	10^th^	25^th^	50th	75^th^	90^th^	95^th^	97.5^th^
LDLC (mg/dl)	126 ± 48	49	60	72	93	120	153	186	212	234

## Discussion

Large numbers of subjects with elevated LDLC fail to achieve treatment targets [7–9], for multiple reasons including statin intolerance [10, 11], expense, lack of insurance coverage, or variations in statin availability across states in insurance, race, and ethnicity [7]. Beyond statin effects, the PCSK9 inhibitors now offer the promise of optimizing LDLC in a majority of patients with HeFH, CVD, and statin intolerance [2, 5, 12–15]. The issue of healthcare systems either facilitating or hindering optimal delivery of hyperlipidemia management will now be amplified by the paradigm-shifting improvement in LDLC lowering by the PCSK9 class of medications [1, 2, 16] at an annual price of $14,000–14,600 per patient.

In the current study, of the 734 patients referred to a Lipid Center for diagnosis and treatment of high LDLC and/or CVD, with LDLC ≥ 70 mg/dl despite maximally tolerated cholesterol lowering therapy, 30 % were eligible by FDA [17] and insurance carrier criteria for PCSK9 inhibitor therapy. Our analysis is focused on those high risk patients for whom PCSK9 inhibitors are specifically indicated [1, 2, 17] and restricted by medical insurance carriers, as an adjunct to diet-maximally tolerated cholesterol lowering therapy in adults with HeFH, and/or CVD who require additional lowering of LDLC. As for the general population, the recent CDC report on cholesterol treatment in the general population of the US [3] found that 36.7 % (78 million) adults (> 21 years) were eligible for cholesterol-lowering medication, but of this group, only 55 % were actively taking a cholesterol lowering medication (~90 % a statin drug). If 30 % of the 78 million hypercholesterolemic adults in the general US population [3] were, as in our current study, eligible by FDA [17] and insurance carrier criteria for PCSK9 therapy, this would include 11 % of the adult population or 23.4 million adults.

The population cost of PCSK9 inhibitor therapy largely depends on the number of subjects judged to be at high risk by virtue of HeFH with high LDLC [18–20], and/or by CVD with LDLC above target goals [4] despite maximally tolerated therapy. In 1.3 million adults, Elshazly et al. [18] identified the 97^th^ percentile of LDLC as 190, 160–190 as the 90^th^–97^th^ percentile, and 130–160 mg/dl the 72^nd^–90^th^ percentile. In our 8053 patients referred for diagnosis and therapy of hypercholesterolemia, 9 % had LDLC ≥ 190 mg/dl and 21 % had LDLC ≥160 mg/dl. Hence, up to 21 % of our hypercholesterolemic referrals had LDLC levels usually associated with HeFH, making them good candidates for approval [17] of PCSK9 therapy by commercial insurance carriers. Toth et al. [20] examined NHANES 2005–2006 data, and identified 27 % with high LDLC (risk stratum-specific). In studies from the US general population 2009–2010 NHANES survey, Fryar et al. [19] identified “uncontrolled high LDLC (≥ 130 mg/dl)” in 28 % of adults. Kuklina et al. have estimated that more than one-quarter of adults aged 40–74 have high LDLC [21]. If 21–36.7 % [3, 19–21] of the general adult US population is judged to have high LDLC, as above, then a high LDLC cohort would range from 45 to 78 million subjects. Extrapolating from our referral cohort where 30 % of hypercholesterolemic patients had HeFH and/or CVD with LDLC ≥100 mg/dl despite maximally tolerated cholesterol lowering therapy, it is possible that 13 to 23 million patients might be candidates for treatment with PCSK9 inhibitors.

Direct costs of CVD include those related to the diagnosis and treatment of the condition, while indirect costs include lost work productivity, loss of future productivity, unemployment, or death [22]. In 2010, CVD related direct medical costs in the United States were estimated to be $273 billion, and indirect costs 172 billion, $445 billion total [23]. The American Heart Association estimated that direct and indirect costs of medical care for CVD approached $450 billion in 2010, and are projected to rise to over $1 trillion by 2030 [24]. Heidenrich et al. [23] have projected that by 2030, 40.5 % of the US population may have some form of CVD. From 2010 to 2030, real total direct medical costs of CVD are projected to triple, from $273 billion to $818 billion. Real indirect costs (due to lost productivity) for all CVD are estimated to increase from $172 billion in 2010 to $276 billion in 2030, an increase of 61 % [23]. Programs targeted to prevention of CVD should provide substantial overall cost savings [25, 26].

Given current pricing of $14,000–14,600 per patient per year, annual PCSK9 inhibitor costs might approximate $185–$342 billion, reflecting the use of a currently expensive drug for an endemic disease, CVD, the leading cause of mortality in the USA [23, 27]. In 2011 the annual costs for CVD and stroke were estimated to be $320.1 billion [24]. If, speculatively, CVD incidence could be halved by PCSK9 therapy [1, 2, 16], direct annual savings would be estimated to be $160 billion, and indirect annual savings might be $85 billion [23], altogether $245 billion savings, in the middle of the range of estimated PCSK9 inhibitor costs of $185–342 billion.

Whether and to what degree PCSK9 inhibitor use will facilitate overall reductions in the cost to society will depend on how widely they will be used, and the percentage reduction of all cause and CVD mortality [28]. Overall costs to society also need to include analysis of any potential adverse effects arising from PCSK9 inhibitor use. On Alirocumab, injection site reactions, myalgia, neurocognitive events, and ophthalmologic events were more common than on placebo [1]. On Evolocumab, nonspecific adverse events (arthralgia, headache, limb pain, fatigue, and neurocognitive events) were reported more frequently than in the placebo group [2]. In a meta-analysis of 25 randomized controlled trials with PCSK9s, Zhang et al. [16] “detected largely no significant difference in major adverse events rates between antibody administration and control treatment, and no difference between different dosages of Evolocumab.” Within this frame of reference, we speculate that costs to society arising from adverse effects specifically attributed to PCSK9 inhibitors may be minimal.

Changes in lifestyle (diet and exercise), to the extent that they lower CVD event rates [29–31], may play a role in the cost-benefit analysis by further reducing CVD event rates in concert with PCSK9 therapy [28].

When statin medications were introduced, cost of illness calculations were made [32] with the conclusion that “When only direct medical care costs were considered, the incremental cost-effectiveness ratios for lifelong therapy with atorvastatin calcium, 10 mg/day, were generally positive, ranging from a few thousand to nearly $20,000 per year of life saved”. When the societal point of view was adopted and indirect costs were included, the total costs were generally negative, representing substantial cost savings (up to $50,000 per year of life saved) and increased life expectancy for most groups of individuals. Grover et al. [32] concluded that adding the indirect CVD costs associated with productivity losses at work and home can result in forecasted cost savings to society as a whole such that lipid therapy could potentially save lives and money. Cost effectiveness was also estimated from the use of Crestor (20 mg/day) in the JUPITER trial [33], and it was concluded that the treatment was cost-effective for higher risk patients, Framingham CVD risk ≥ 10 %. Song et al. [22] reported that prevention or reduction in CVD events could result in cost-savings for employers.

Strengths of our study include documentation that, of patients referred to a regional center for therapy of high LDLC, 30 % had HeFH and/or CVD, and despite maximal LDLC lowering therapy, retained LDLC > 100 mg/dl, meeting both FDA indications [17] and commercial insurance coverage eligibility. We also evaluated a group of 50 patients approved for and already receiving insurance coverage for PCSK9 inhibitor therapy, who had HeFH and/or CVD, whose suboptimal LDLC lowering despite maximal lipid lowering therapy, emphasizes the medical necessity of PCSK9 inhibition in high risk patients. It is also noteworthy that within the 50 approved patients, there were five with LDLC in the 80–97 mg/dl range. Limitations include bias towards higher LDLC and CVD by virtue of referral to a regional Cholesterol Treatment Center, which would increase the percentage of hypercholesterolemic patients eligible for insurance coverage. However, in the US general population, the CDC has estimated that 36.7 % of adults should be eligible for cholesterol lowering medications [3], with an LDLC range similar to our 734 patients referred to us for diagnosis and treatment of hypercholesterolemia.

## Conclusions

Although the costs for PCSK9 inhibitors when given to an estimated 13 to 23 million patients would be extraordinary ($185–342 billion), we speculate that if there was a 50 % reduction in CVD, then there would be savings of $245 billion, in the middle of the range of estimated PCSK9 inhibitor costs of $185–342 billion. Whether the health care savings arising from the anticipated reduction of CVD on the PCSK9 inhibitors justify the broad population use of these agents remains to be determined.

## Abbreviations

CVD: cardiovascular disease
HeFH: heterozygous familial hypercholesterolemia
LDLC: low density lipoprotein cholesterol
